# Supplementary material for: Modeling glioblastoma heterogeneity as a dynamic network of cell states
Source: Mol Syst Biol. 2021 Sep 16;17(9):e10105. doi: 10.15252/msb.202010105 (PMC8444284; doi:10.15252/msb.202010105)
Supplement: Supplementary file 6 — Source Data for Figure 5 [file MSB-17-e10105-s004.zip › Figure5A_sourcedata/GSEA_3017/hallmarks_stateF.GseaPreranked.1621934628066/HALLMARK_UV_RESPONSE_DN.html]

Details for gene set HALLMARK\_UV\_RESPONSE\_DN[GSEA]

|  || Dataset | state33017 |
| Phenotype | NoPhenotypeAvailable |
| Upregulated in class | na\_pos |
| GeneSet | HALLMARK\_UV\_RESPONSE\_DN |
| Enrichment Score (ES) | 0.32749885 |
| Normalized Enrichment Score (NES) | 1.5234417 |
| Nominal p-value | 0.051457975 |
| FDR q-value | 0.0788539 |
| FWER p-Value | 0.694 |
Table: GSEA Results Summary

  

Fig 1: Enrichment plot: HALLMARK\_UV\_RESPONSE\_DN      
 Profile of the Running ES Score & Positions of GeneSet Members on the Rank Ordered List

  

| PROBE | GENE SYMBOL | GENE\_TITLE | RANK IN GENE LIST | RANK METRIC SCORE | RUNNING ES | CORE ENRICHMENT || 1 | F3 |  |  | 41 | 0.978 | 0.0500 | Yes |
| 2 | LTBP1 |  |  | 108 | 0.741 | 0.0721 | Yes |
| 3 | ANXA4 |  |  | 127 | 0.703 | 0.1131 | Yes |
| 4 | ATP2B1 |  |  | 170 | 0.632 | 0.1384 | Yes |
| 5 | COL5A2 |  |  | 199 | 0.589 | 0.1670 | Yes |
| 6 | PDLIM5 |  |  | 223 | 0.572 | 0.1967 | Yes |
| 7 | SCHIP1 |  |  | 233 | 0.566 | 0.2322 | Yes |
| 8 | PIK3R3 |  |  | 251 | 0.553 | 0.2633 | Yes |
| 9 | ATP2B4 |  |  | 356 | 0.485 | 0.2503 | Yes |
| 10 | GJA1 |  |  | 495 | 0.436 | 0.2187 | Yes |
| 11 | TFPI |  |  | 531 | 0.421 | 0.2324 | Yes |
| 12 | IGFBP5 |  |  | 564 | 0.410 | 0.2466 | Yes |
| 13 | SDC2 |  |  | 596 | 0.399 | 0.2606 | Yes |
| 14 | ADD3 |  |  | 674 | 0.378 | 0.2524 | Yes |
| 15 | PRKAR2B |  |  | 707 | 0.369 | 0.2637 | Yes |
| 16 | VLDLR |  |  | 713 | 0.368 | 0.2873 | Yes |
| 17 | SIPA1L1 |  |  | 717 | 0.367 | 0.3116 | Yes |
| 18 | MAP1B |  |  | 762 | 0.355 | 0.3166 | Yes |
| 19 | ATRX |  |  | 793 | 0.348 | 0.3275 | Yes |
| 20 | BHLHE40 |  |  | 928 | 0.321 | 0.2897 | No |
| 21 | DLG1 |  |  | 1013 | 0.307 | 0.2733 | No |
| 22 | ATRN |  |  | 1037 | 0.302 | 0.2841 | No |
| 23 | KALRN |  |  | 1106 | 0.292 | 0.2739 | No |
| 24 | ATXN1 |  |  | 1138 | 0.286 | 0.2800 | No |
| 25 | PLPP3 |  |  | 1213 | 0.277 | 0.2660 | No |
| 26 | ICA1 |  |  | 1394 | 0.257 | 0.2030 | No |
| 27 | NOTCH2 |  |  | 1420 | 0.255 | 0.2095 | No |
| 28 | CITED2 |  |  | 1433 | 0.254 | 0.2219 | No |
| 29 | AGGF1 |  |  | 1454 | 0.252 | 0.2305 | No |
| 30 | INPP4B |  |  | 1456 | 0.251 | 0.2477 | No |
| 31 | ANXA2 |  |  | 1511 | -0.263 | 0.2418 | No |
| 32 | HAS2 |  |  | 1968 | -0.464 | 0.0689 | No |
| 33 | SERPINE1 |  |  | 2161 | -0.843 | 0.0414 | No |
Table: GSEA details [plain text format]

  

Fig 2: HALLMARK\_UV\_RESPONSE\_DN: Random ES distribution      
 Gene set null distribution of ES for **HALLMARK\_UV\_RESPONSE\_DN**
